# Electroplated double-crowns on implants and teeth after up to 12 years– a retrospective clinical study

**DOI:** 10.1186/s40729-025-00594-x

**Published:** 2025-02-03

**Authors:** Christoph Mautsch, Jan Klenke, Thomas Kern, Stefan Wolfart, Jaana-Sophia Kern

**Affiliations:** 1 Department of Prosthodontics and Biomaterials, Center for Implantology, Medical Faculty, RWTH Aachen University, Pauwelsstr. 30, 52074 Aachen, Germany; 2 Private Dental Practice, Große Bleichen 32, 20354 Hamburg, Germany

**Keywords:** Telescopic crowns, Double-crowns, Galvano-crowns, Electroplating, Removable dental prosthesis, Implant-supported, Clinical study, Electro-plated crowns

## Abstract

**Purpose:**

To retrospectively evaluate the outcome of implant-supported or combined tooth-implant-supported prostheses retained by electroplated double-crowns after 1–12 years.

**Methods:**

Twenty-five patients were retrospectively examined in a private dental practice in Hamburg, Germany. All had been rehabilitated with a removable prosthesis retained by electroplated double-crowns, for at least one year. Fifteen patients had implant-supported prostheses and 10 had combined tooth-implant-supported prostheses in the maxilla or the mandible. Biological and technical complications were recorded at the clinical examination and extracted from the patient records. Kaplan–Meier implant and tooth survival rates were calculated. Potential risk factors for severe complications were identified. Oral health-related quality of life (OHRQoL) was measured by a short version of the Oral Health Impact Profile (OHIP) questionnaire. Patients reported subjective chewing function using a visual analogue scale.

**Results:**

Kaplan–Meier survival rates were 100% for natural abutments and 90.9% for implants after 11.8 years (p = 0.54). Two implants in two patients were lost at 8 and 9 years due to peri-implantitis in the "solely implant" group. The most common complications were decementation of primary crowns and wear of the prosthetic teeth. The mean OHIP score for the group “tooth-implant-supported” was 5.2 ± 5.0, whereas the mean score for the "solely implant" group was 1.7 ± 2.9 (p = 0.039). Patients rated their subjective masticatory function very high with an average score of 9.4 ± 0.8 out of a possible 10.

**Conclusions:**

Implant-supported or combined tooth-implant-supported prostheses retained by electroplated double-crowns are a viable method of treatment with a satisfactory outcome. Maintenance has been manageable and patients have reported very good subjective chewing function after several years of function.

**Graphical Abstract:**

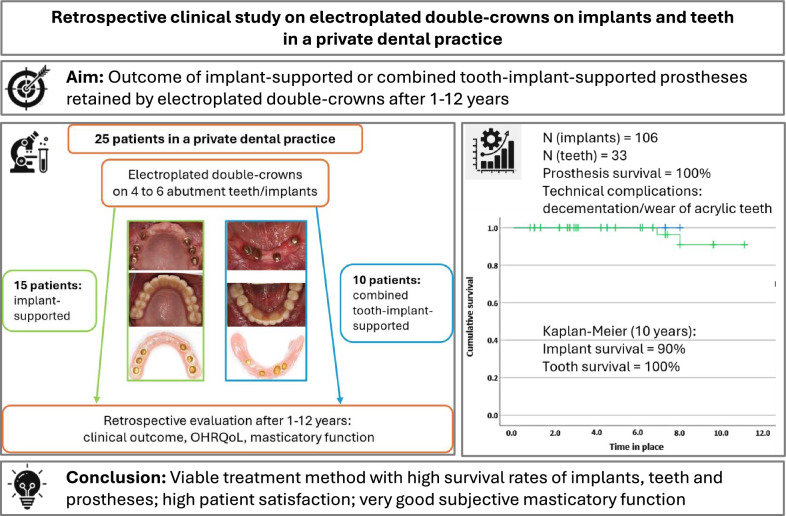

## Background

Removable dental prostheses being retained by double-crown attachments are based on the principle of primary crowns cemented to the abutment teeth and secondary components connected to the prosthesis. They are successfully used to rehabilitate partially or completely edentulous jaws [1, 2] although with regard to survival rates of abutment teeth, a wide range (60.6–100% after 4–10 years) is to be observed [3, 4]. Double-crown systems, in contrast to fixed restorations or clip-retained prostheses, offer the advantage that also compromised teeth with uncertain prognosis can be included as abutments, as repair and extension measures are very easy to handle. While providing a feeling of fixed teeth for the patient, double-crown retained prostheses still offer optimal conditions for oral hygienic measures. Severely reduced dentition can be supported by strategically placed implants as a symmetrical distribution of abutments can reduce the failure rate of natural abutments and contribute to the stability of the reconstruction [5, 6] and the combination of teeth and implants appears to be a viable method for attaching double-crown retained prostheses [7–12]. In this context, a recent meta-analysis calculated an estimated survival rate of 98.8% for implant abutments and 95.4% for natural abutments with telescopic crowns [13]. Especially when using double-crowns and combining implants and teeth, the use of electroplated ("galvanic") crowns can be advantageous.

The special fabrication method of these crowns enables an intraoral luting of secondary crowns and framework, which leads to a very accurate, passive fit, even if a combination of “mobile” teeth and rigid implants is used for the attachment.

While there is currently some data available on the use of electroplated double-crowns [10, 11, 14–16], the number of studies remains limited with regard to electroplated double-crowns and the combination of implants and teeth. The majority of studies on electroplated double-crowns were conducted in academic research settings [10, 11, 14–16], and only two studies used precious metal alloy instead of ceramics for the primary crowns [14, 15].

With regard to the OHRQoL, Stober et al. and Liebermann et al. have demonstrated an improvement following treatment with double-crowned retained prostheses [17, 18]. However, further data from clinical long-term studies are still required to confirm these findings. This also applies to the subjective chewing ability associated with this type of prosthesis. The occurrence of complications such as acrylic fractures, chippings, endodontic or implant-related problems can be stressful for both the dentist and the patient as it involves additional visits to the dental office. Manufacturing-related and expected retention loss of double-crown prostheses [19] often takes several years to appear. A review of the data on technical complications from the existing clinical studies, which were also summarized by Moliner-Mourelle et al., reveals that the most common complications are screw loosening, veneering fractures, and, less frequently, the need for recementation [13]. This appears to be independent of the number of implants/natural abutments [13]. Biological complications were more likely to be moderate to severe compared to technical complications. These included, for instance, peri-implant infections, caries, and pulpitis, but also implant and tooth loss [13].

The aim of this retrospective clinical study was to assess the clinical outcomes of implant-supported and combined tooth-implant-supported removable prostheses retained by electroplated double-crowns with primary crowns from precious metal alloy after 1 to 12 years in a private dental practice. Kaplan–Meier survival rates for implants and abutment teeth were calculated and the overall success of the prostheses was analyzed. Additionally, we evaluated the OHRQoL and subjective masticatory function of patients with these types of prostheses.

## Methods

### Trial design

This retrospective clinical cohort study was approved by the Ethics Committee (EK Nr. 123/15) of the Medical Faculty of RWTH Aachen University and was conducted following the ethical standards of the Declaration of Helsinki. Clinical follow-up was performed by authors JSK, TK, and a dental assistant in a private dental practice in Hamburg (Germany) between May 2015 and June 2016. The study was registered in the German Clinical Trials Register (DRKS00033746; date of registration 29/02/2024).

### Patients

**Fig. 1:**
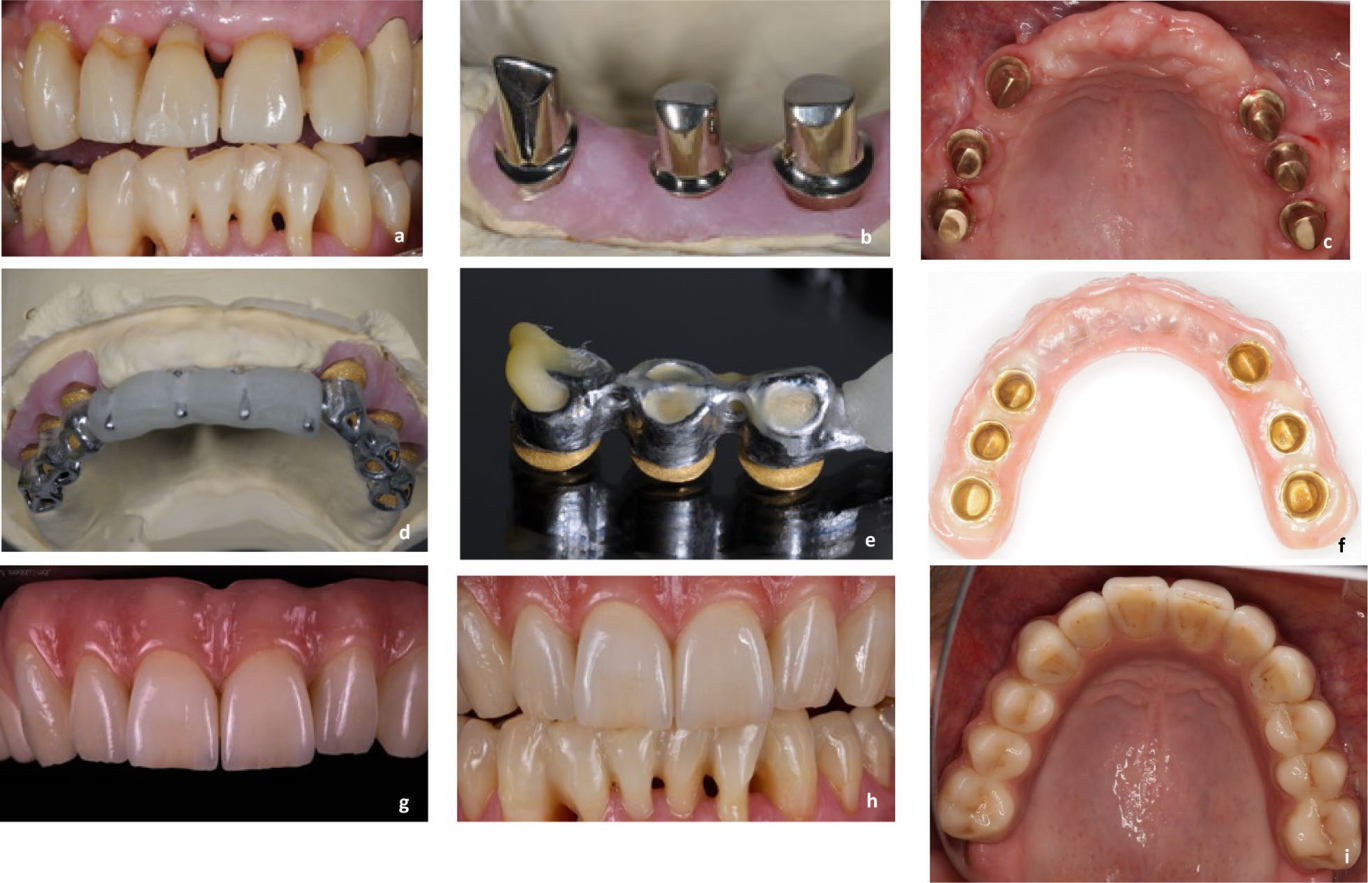
**a-i** A clinical example case of the study. **a**. Initial situation in the maxilla with still existing hopeless teeth. **b** Three of the primary crowns on the master cast. **c**. Situation after placement of 6 implants and cementation of primary crowns. **d** Master cast with electroplated secondary crowns and the tertiary framework made of cobalt-chromium alloy. **e** Three of the electroplated secondary crowns after intraoral adhesive luting to the framework. **f** Basic view of the finished prosthesis. **g** Front view of the finished prosthesis. **h** Front view of the finished prosthesis in situ. **i** View of the maxilla with the prosthesis in place at the time of the follow-up examination

All patients with an implant-supported or combined tooth-implant-supported electroplated double-crown removable dental prosthesis (ED-RDP) (Fig. 1a–f) were invited to participate in this retrospective study as part of their regular follow-up.

The inclusion criteria were.

- prosthesis in place for at least one year
- minimum age of 18 years
- a general medical condition that allowed a clinical dental examination
- informed consent was given prior to inclusion

Exclusion criteria were.

- pregnancy
- inability to give informed consent
- any reason contraindicating a clinical dental examination (e.g. severe psychological disorders)

### Prosthodontic treatment

The patients who were investigated in this study had experienced extensive tooth loss in the past and a fixed reconstruction was not an option. Following a joint decision with the patients, it was agreed that an ED-RDP should be incorporated on implants or a combination of implants and teeth. Remaining teeth were conservatively pre-treated where necessary. The aim was to have at least six abutments (implants/and or natural) in the maxilla and four in the mandible.

Most of the patients had a few remaining but hopeless teeth that had to be extracted (Fig. 1a). In these cases six implants were inserted into the upper and/or four into the lower jaw (Fig. 1b). Other patients had one to four teeth that could be retained, and additional strategically positioned implants were inserted to achieve a quadrangular support of the prosthesis.

Implant surgery was performed by author JK and two maxillofacial surgeons in 23 patients, and two patients had already received implants *alio loco*. Bone augmentation and maxillary sinus membrane elevation were performed prior to implant placement whenever necessary. An allogeneic bone graft material (Bio-Oss, Geistlich Pharma AG, Switzerland) was used in these cases. The majority of patients received conical, self-tapping Camlog or Conelog Screw-line implants (Camlog Biotechnologies GmbH, Basel, Switzerland). One patient received cylindrical Straumann Bone Level implants (Straumann GmbH, Basel, Switzerland). After 4 to 6 months of submerged healing, the implants were uncovered and temporary gingiva formers were placed. Natural abutment teeth were prepared with a 2 mm circumferential reduction and a pronounced chamfer. Temporary restorations were placed. After a further healing period of approximately 6 weeks, an impression of the implants (and prepared teeth, if any) was taken with a polyether material (Impregum, 3 M Deutschland GmbH, Neuss, Germany) and an open custom impression tray. The primary crowns were fabricated using the classic method (wax-up technique, cast in a gold alloy, milled to a convergence angle of 0°, Fig. 1b). After the try-in of these primary crowns, a fixation impression was taken together with implant impression posts, and the inner copings for the implants were fabricated. The secondary crowns were then electroplated directly onto the primary crowns. The prosthetic framework, also called the tertiary framework, was manufactured from a cobalt-chromium alloy. After checking the accuracy of fit of all components, the screw-retained implant abutments were definitively placed and the primary crowns were cemented to all abutments (implants and teeth, Fig. 1c) with a Zinc-phosphate cement (Harvard cement, Harvard Dental International, Hoppegarten, Germany). The electroplated secondary crowns were then cemented intraorally into the tertiary structure with an autopolymerizable luting composite (AGC Cem, Wieland Dental, Pforzheim, Germany) to achieve an optimal “passive” fit (Fig. 1d, e). A second registration of the maxillomandibular relationship with the incorporated framework was performed, followed by another fixation impression. Patients then received a new provisional prosthesis to cover the primary crowns. The final superstructures were completed in the dental laboratory and placed in the patients' mouths (Fig. 1f–i). Author JK performed the prosthetic treatment for all patients. Patients were seen two to four times per year for check-up appointments and professional dental cleaning.

### Follow-up examinations

All investigators were calibrated prior to the study. After at least one year of wearing, patients were clinically examined by authors JSK, TK and a dental assistant from the private practice between May 2015 and June 2016. Patients were asked about their medical history and were examined extraorally. The intraoral examination included the following parameters.

- Modified plaque and gingiva index [20, 21].
- Probing depth at four sites (distal, buccal, mesial, lingual/palatal) with a periodontal probe
- Recording of biological and technical complications associated with minimal (1), moderate (2) or extensive (3) treatments, such as
- (1) Treatment of pressure sores, occlusal adjustments, relining, retightening or insertion of new abutment screw
- (2) Fillings, root planing/periodontitis therapy, peri-implantitis therapy, primary crown recementation, fracture repair (acrylic parts), acrylic tooth renewal
- (3) Endodontic treatment, tooth or implant removal, post and core revision due to fracture, remake of framework due to fracture.

Peri-implant health was defined according to Berglundh et al. [22]: crestal bone level changes, absence of erythema/bleeding on probing/swelling/suppuration. Peri-implant mucositis was defined as follows [22]: bleeding on gentle probing, inflammation limited to the mucosa.

Additionally, patients completed a German short form of the OHIP (OHIP-G14) on OHRQoL.

The OHIP-G14 included specific items concerning psychic, physical and social limitations and discomfort as well as pain related to the dental prosthesis, which were answered on a 5-point Likert scale ranging from 0 = "never" to 4 = "very often". Higher scores indicate poorer OHRQoL.

Subjective masticatory function was assessed using a visual analog scale (VAS). It covered different types of food (soft to hard consistency, e.g. bread, meat, carrots), with 0 (far left end of the scale) meaning "cannot chew at all" and 10 (far right end of the scale) meaning "can chew without problems".

### Statistics

Statistical analysis was performed using IBM SPSS (version 29, IBM). Descriptive statistics included means, standard deviations, and frequencies for all study parameters. Depending on the specific question, additional analyses were performed at the patient and abutment (tooth/implant) level. Kaplan–Meier analysis was used to calculate cumulative survival and success rates of abutments and prostheses. The severe complications”tooth or implant loss” and “endodontic treatment” were used to calculate success rates. To identify possible risk factors, the occurrence of “severe complications” was plotted against the parameters "age", "type of treatment", "number of abutments", and "type of antagonist treatment" using Chi square test. Furthermore, the dichotomous parameters "gender" and "study jaw" were compared. Patients < 65 years and ≥ 65 years, patients with solely implant-supported prostheses and combined tooth- and implant-supported restorations, restorations on a maximum of 4 abutments and on more than 5 abutments, and patients with fixed or removable restorations in the opposing jaw were compared. These potential variables were plotted against the dichotomous parameter "severe complications" and tested for dependence using the Chi-square test. Additionally, the occurrence of different complications was examined in general and within the same subgroups. After testing for normal distribution using the Shapiro–Wilk test, the mean values of the subgroups were compared using the Mann–Whitney U test. When analyzing the clinical parameters "probing depths", “gingiva index" and "plaque index", only the highest index value per tooth/implant was considered. Mean comparisons were made between the subgroups described above in the same manner. In addition, the results were compared between natural abutments and implants. Means, standard deviations, and medians were calculated for both the OHIP total score and the individual questions. Analogous to this, an evaluation of the chewing ability of the different foods was carried out. As a global parameter, a patient-specific mean value for the masticatory function was calculated from the sum of the individual ratings. Subgroup comparisons were performed according to the previously described procedures. In addition, a linear relationship was tested between the parameters "OHIP", "masticatory function", "observation time" and "number of complications". Due to the non-normal distribution of the data, the Spearman-Rho correlation test was applied.

## Results

### Included patients

**Fig. 2:**
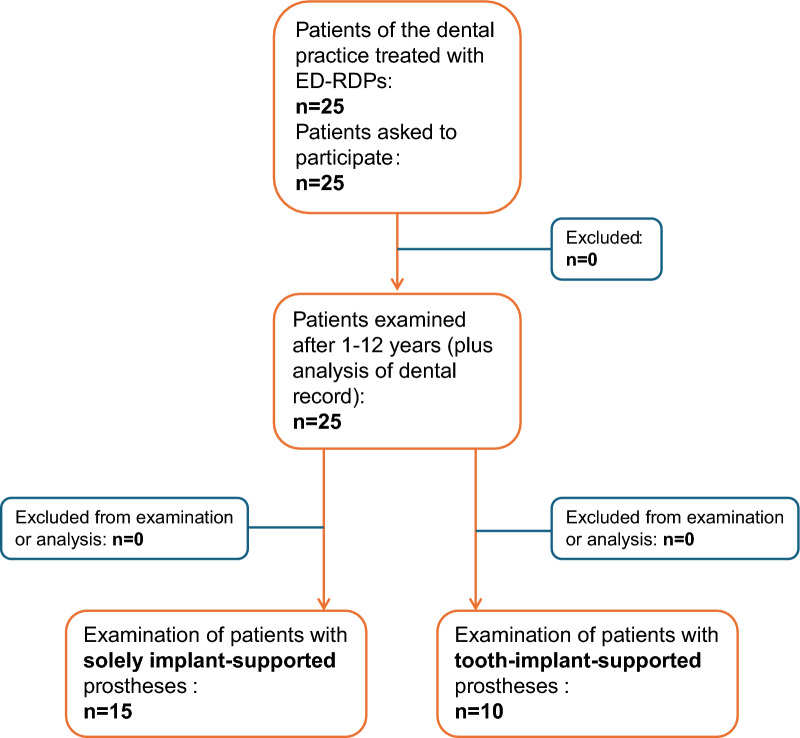
Patient flow chart. Overview of included patients

Twenty-five patients (mean age 68.4 ± 9.9 years, 60% female) with 25 ED-RDPs with a mean wearing period of 4.9 ± 3.0 years were examined during the period from May 2015 to April 2016. Seventeen of the prostheses were in the maxilla and eight in the mandible. In total, 139 abutments were used for the electroplated double-crowns. These included 106 implants and 33 natural abutments. Fifteen restorations were solely implant-supported (n implants = 81), and ten restorations were tooth-implant supported (n (implants) = 45, n (teeth) = 33) (Fig. 2).

### Survival and success analysis

All 106 implants were included in the analysis. As all patients originated from the same practice and represent all patients who have ever received an ED-RDP at this practice, it can be assumed that no data loss has occurred, including instances of unregistered implant loss.

**Fig. 3:**
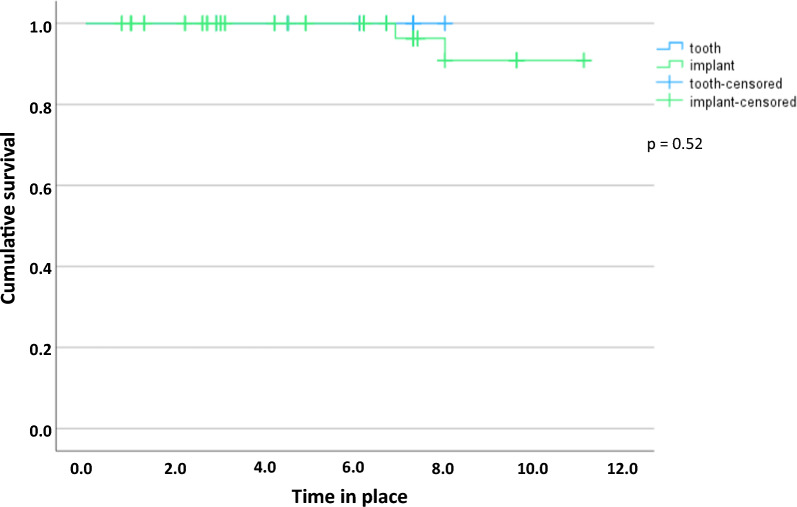
Cumulative implant survival after 11.8 years according to Kaplan–Meier analysis. Two implants were lost at 8 and 9 years. No abutment tooth was lost

With a minimum of 1.3 years and a maximum of 11.8 years, the mean time in place of the implants was 5.2 ± 3.1 years. Altogether two maxillary implants were lost in two patients after 8 and 9 years due to peri-implantitis in the group with solely implant-supported RDPs. This corresponds to a cumulative post-loading implant survival rate of 90% at 10 years according to the Kaplan–Meier analysis (Fig. 3). Early failures, i.e. implant losses before loading, did not occur. The survival rate of the natural abutments was 100%.

**Fig. 4:**
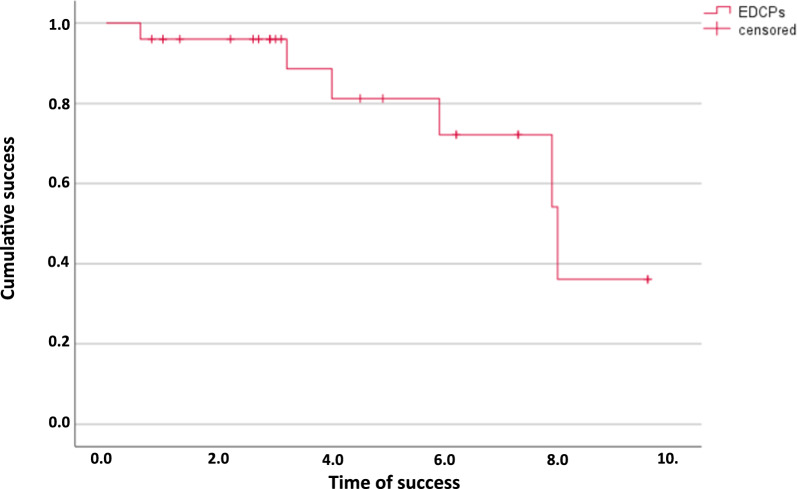
Cumulative prosthesis success after 9.6 years according to Kaplan–Meier analysis. A total of 6 prostheses experienced complications (4 endodontic treatments, 2 implant losses) that compromised the success of the prostheses. None of the prostheses required replacement

Prosthesis survival was 100%. At follow-up, none of the prostheses had been replaced. A total of six of the 25 RDPs (24%) were associated with at least one severe complication (implant loss or irreversible pulpitis) and corresponding Kaplan–Meier cumulative success rates were 81% at 5 years and 36% at 8 years (Fig. 4). The first observed major complication occurred after 0.6 years. The longest observation period without major complications was 9.6 years. On average, the prostheses had been successfully in use for 4.3 ± 2.8 years, which means that no major complications had occurred up to that point.

### Technical and biological complications

Table 1 provides an overview of all recorded technical and biological complications which had occurred after prosthesis placement. The most common technical complication was decementation of the primary crowns (n = 13) and wear of the prosthetic teeth (n = 11), followed by nine cases of necessary relining.

**Table 1:** Technical and biological complications associated with minimal (1), moderate (2) or extensive (3) treatment since prosthesis placement

Treatment level	Type of complication	Number of complications	Percentage of total complications (%)
1	Relining necessary	9	13.0
	Exposed metal framework	1	1.4
	Prosthesis stomatitis	6	8.7
2	Decementation of primary crown	13	18.8
	Wear of prosthetic teeth	11	15.9
	Fracture of prosthetic teeth/chipping	8	11.6
	Gingivitis/mucositis	14	20.3
3	Irreversible Pulpitis of natural abutment	3	4.3
	Fracture of natural abutment	1	1.4
	Implant loss	2	2.9
	Tooth loss	0	0
**Total**		**69**	**100**

Gingivitis or mucositis was observed in 14 cases. Two implants were lost after eight and nine years. They had previously shown signs of peri-implantitis. These cases, along with four cases requiring endodontic treatment (in one case followed by post-and-core treatment) were considered severe complications and were used to calculate the success rate.

With the help of the Chi-square test, the number of abutment teeth could be identified as a risk factor for the occurrence of severe complications. While only two out of 19 (10.5%) subjects with five or more abutments experienced severe complications, four out of six (66.7%) subjects with a maximum of four abutments experienced severe complications (p = 0.005). An analysis of all complications also showed an increased incidence in patients with a maximum of four abutments. While subjects with five or more abutments had an average of 2.4 different complications, the other group had an average of 4.3. However, this difference was not statistically significant.

No association was found between the other potential risk factors "age", "location", "gender", "type of opposing dentition" and "solely implant-supported vs. combined tooth-implant-supported" and the occurrence of severe complications.

### Probing depths, gingiva and plaque index

The mean probing depths were 3.0 ± 0.9 mm for the natural abutments and 3.7 ± 1.3 mm for the implants. The mean values of the gingival and plaque indices were 1.3 ± 0.8 and 1.1 ± 0.8 for natural teeth and 1.2 ± 0.7 and 1.1 ± 1.0 for implants, respectively. While the values for gingival and plaque index were comparable for teeth and implants, the implants showed a statistically significant higher probing depth than the natural teeth (p = 0.008). The subgroup comparison of these clinical parameters revealed a significantly higher plaque index in the group of patients ≥ 65 years of age (p = 0.049) and on prostheses with a maximum of four abutments (p = 0.001). An increased probing depth could be observed in male patients (p = 0.018) and in the upper jaw (p = 0.018). Of the six patients with prostheses on a maximum of four abutments, five were in the " ≥ 65 years" group. However, a statistical relationship between the parameters "age" and "number of abutments" could not be established.

### Oral-health related quality of life

As part of the follow-up for this study, the patients' OHRQoL was assessed using the OHIP-G 14. One of the patients did not fill in the OHIP-questionnaire. The mean OHIP total score of the patients was 3.2 ± 4.2. The sum scores ranged from 0 to 13 with a median of 1. Eleven of the 24 patients (45.8%) did not report any limitations, while another eight patients reported a maximum OHIP sum score of 4, indicating only a slight reduction in quality of life. Slight limitations were observed in five of the 24 respondents. Two patients reported frequent to very frequent difficulties with word articulation. In addition, two patients reported that their life had become generally less satisfying in recent times in connection with their prostheses. Another patient reported very frequent pain in the oral cavity, but otherwise did not report any reduction in quality of life.

In a comparison of the subgroups, there are more reports of impairments in the group with combined tooth-implant supported ED-RDPs. The mean OHIP score for this group was 5.2 ± 5.0, whereas the mean score for the "solely implant" group was 1.7 ± 2.9. This difference was statistically significant (p = 0.039). The comparison of the other subgroups according to the parameters "age", "gender", "study jaw", "number of abutments" and "opposing jaw" showed no significant differences in the OHIP total value. Furthermore, no correlation was identified between the observation period and the OHIP sum score.

### Subjective masticatory function

**Fig. 5:**
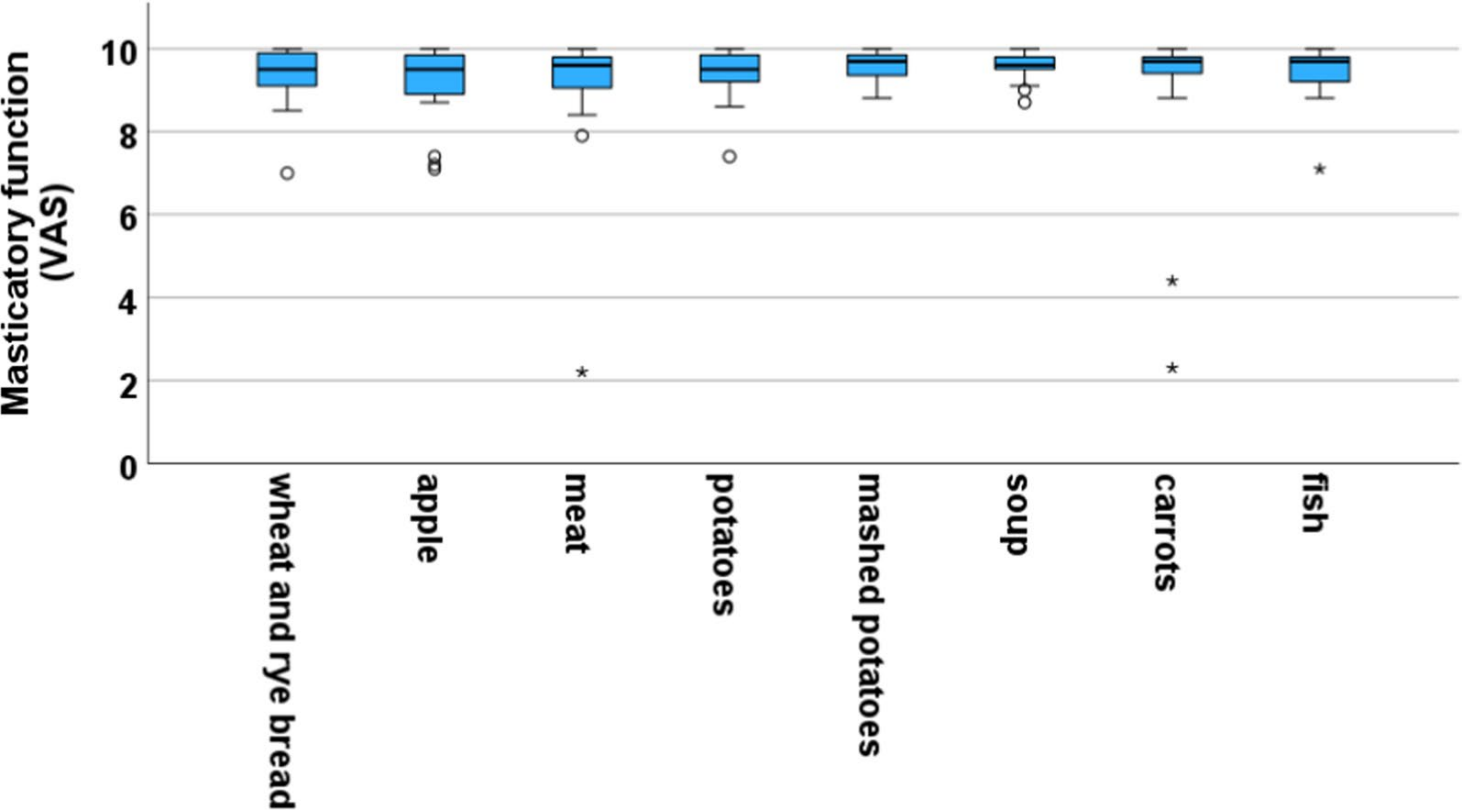
Boxplot analysis of masticatory function as reported by patients using a visual analogue scale (VAS). A point value of 10 meant an unrestricted masticatory function

In terms of subjective masticatory function, the average score was 9.4 ± 0.8 out of a possible 10. Figure 5 gives an overview of the corresponding box plot analysis. Only four of the respondents scored below 9, with one outlier scoring an average of 6.5. In the individual categories, the chewing function was rated worst for carrots (9.1 ± 1.9) and meat (9.1 ± 1.6). With ratings of 2.3 and 2.2, the "outlier" here indicated considerable difficulty in chewing carrots and meat. The subgroup comparison according to the above mentioned parameters showed no statistically significant differences in the masticatory function.

The correlation analysis showed statistically significant negative correlations between the subjective masticatory function and the OHIP total score (p = 0.043), as well as between the number of complications and the general masticatory function (p = 0.017). There was no negative correlation between “prosthesis age/time in function” and masticatory function.

## Discussion

Solely implant-supported or combined tooth-implant-supported ED-RDPs showed satisfactory clinical results, and all of the prostheses were still functioning at the time of the follow-up. Patients attended regular follow-up visits and professional teeth cleaning appointments. In particular, with regard to the potential correlation between RDPs and peri-implantitis, these systematic follow-up appointments seem to be of high importance [23].

When severe complications were included in the calculation of a Kaplan–Meier success rate for the prostheses, the results were 81% at five years, which is comparable to the results of a recent study by Klotz et al. [10], and 36% at eight years. The authors of the mentioned study, however, employed broader criteria to calculate their success rate. In contrast, the rather low cumulative success rates observed in our study were based on only six severe complications: two implant losses and four necessary endodontic treatments (one abutment fracture and three cases of irreversible pulpitis). Despite these complications, the prostheses remained functional, and patients experienced only minor limitations. These complications should be seen in the broader context of prosthetic rehabilitation, as higher rates of tooth loss and caries have been observed with, e.g. conventional telescopic-crown prostheses and a combination of natural abutments and implants [12]. RDPs with electroplated double-crowns are complex and costly to fabricate. Nevertheless, they may offer significant advantages (inclusion of compromised teeth, passive fit, high retention) depending on individual patient situations and preferences. In contrast, overdentures with simpler attachments, such as stud or ball attachments, represent a relatively straightforward and cost-effective solution. However, they often necessitate regular maintenance due to the rapid wear of their components, which leads to a decrease in the prosthodontic success rate [24]. Moreover, the efficacy of such solitary attachments depends on specific conditions. For instance, the vertical height loss should only be minimal and the implants should be as parallel as possible. An additional, less expensive alternative exists in the form of implant-supported bar-retained overdentures. Recent clinical data with four to six implants show high implant survival rates of up to 100% after ten years, however, 19 overdentures had to be replaced due to severe wear of teeth and denture base [25, 26].

The cumulative implant survival of 90% and the prosthetic survival rates of 100% observed in this dental practice study are comparable to the results found in studies conducted in university settings [10, 29]. The authors of these university studies report survival rates of 93.3% and 96.2% for solely implant-supported restorations, and 97.7% and 100% for combined tooth-implant-supported restorations after 5 and 8 years, respectively. This suggests that the outcomes of this complex treatment modality are consistent across different types of clinical environments. There were, however, some biological and technical complications (Table 1), but these were usually resolved with little or moderate effort, so that the overall maintenance effort can be considered acceptable. Decementation was the most common technical complication for the primary crowns, all of which were cemented with zinc phosphate cement. Today, the use of zinc phosphate cement is viewed rather critically as its disadvantages, such as poor mechanical properties, outweigh its advantages [27]. A retrospective study found that 75% of the 577 patient cases examined after 15 years showed decementation of primary crowns cemented with either zinc phosphate cement or glass ionomer cement [28]. The results of in vitro studies examining the retention of various cements on implant abutments are, however, inconsistent. Zinc phosphate cement has demonstrated high retention values in some of these studies, indicating that retention is not solely dependent on the cement itself but also influenced by additional factors, including the abutment design, crown material, surface treatment, and the specific study design [29–31].

Our analyses indicated that a number of abutments less than four led to a higher total number of complications and a higher number of severe complications in comparison to the patient group with five or more abutments. As already described for conventional combined or tooth-implant-supported double-crown prostheses, an increased incidence of severe complications could be observed for prostheses on up to four abutments. However, this association has not yet been demonstrated for prostheses retained by electroplated double-crowns [10, 32], and also the aforementioned meta-analysis was unable to identify such a correlation [13]. Other risk factors could not be identified in our study. Peri-implantitis was diagnosed in two cases and the affected implants had to be removed after eight and nine years. Considering the prevalence of peri-implantitis at patient level reported in the current literature (nearly 20%) [33–35], the number of cases recorded in this study was surprisingly low. Mean implant probing depth was 3.7 ± 1.3 mm, however, this value alone cannot provide any information about peri-implant health. The current consensus and accepted practice is that it is not possible to assess peri-implant health based solely on a range of probing depths [22]. Although mucositis could be detected in several cases, the clinical findings did not provide a justifiable indication for radiography, and radiography without a suspected diagnosis was not part of the study protocol. Furthermore, it should be noted, that due to the retrospective nature of the study, no baseline data were available for comparison. The mean probing depth of 3.0 ± 0.9 mm for the natural abutments can be considered to be associated with healthy periodontal conditions. There was no tooth loss associated with the ED-RDPs but endodontic problems requiring root canal treatment occurred in three cases. In their systematic review, Moldovan et al. [36] found very different rates of tooth loss (5.5—51.7%) in double-crown restorations. The rates of necessary root canal treatment and tooth fractures were also highly variable, ranging from 0.6 to 13.9% and 0.4 to 4.4%, respectively.

Patients generally reported few problems with their prostheses and indicated that the prostheses provided very good retention, even after a prolonged wear. In clinical studies, different groups were able to demonstrate the positive influence of double-crown prostheses on OHRQoL [17, 18]. In this study, nearly 50% of patients reported no limitations at all in the OHIP questionnaire. The generally low OHIP scores correlate with high levels of satisfaction. In this context, no differences were observed regarding the duration of prosthesis use. It is, however, noteworthy that patients who received solely implant-supported restorations exhibited greater satisfaction than those who received tooth-implant-supported restorations. Again, due to the retrospective nature of the study, patients were not interviewed before receiving the prosthesis, so it cannot be determined whether the high OHRQoL can actually be attributed to the treatment with an ED-RDP. The majority of patients also rated their chewing function as very satisfactory. Even the ability to eat hard foods such as carrots and apples or relatively hard-to-chew meat was rated an average of 9.1 on the VAS. With regard to masticatory function, many recent studies have compared conventional full dentures with implant-supported dentures in the edentulous mandible and found that both subjective and objective masticatory function improved significantly after implant placement [37–39]. Of course, these results can only be extrapolated to our data to a limited extent because we do not know if or how the number of abutments and the opposing dentition play a role in this context. However, a recent meta-analysis showed that chewing performance correlates with the number of natural teeth and functional tooth pairs, among other factors [40]. In our analysis, we found that worse masticatory function correlated with a higher OHIP sum score. However, further analysis did not show any effect of time in function or age of the prosthesis and subjective masticatory function.

### Limitations

There are some limitations to this study that should be addressed at this point. A retrospective clinical study with a small sample size has clear methodological and practical limitations. Its scientific validity is restricted, especially in terms of causal relationships or ensuring generalizability. Furthermore, the collection of retrospective data is often susceptible to bias, potential incomplete records, and temporal variability, which can further compromise the reliability of the findings. Consequently, interpretation should be approached with caution.

The small sample size of only 25 patients in our study reduces statistical power, increases the risk of random findings, and makes it challenging to detect significant differences or effects. A sample size calculation was not performed.

Individual results and statistical outliers lead to a larger scatter of the results due to their statistical overrepresentation. The duration of wear of the prosthesis partly differed considerably, so that also patients with only one year of function were included in the analysis. An analysis of possible peri-implant bone loss was not possible due to a lack of standardized baseline radiographs. A potential loss of retention of the prosthesis was only asked about and was not measured in an objective way. To enhance the strength of the evidence, larger prospective studies with robust sample sizes and controlled data collection are essential.

## Conclusions

In this retrospective study, ED-RDPs, either combined tooth-implant or implant-supported, showed satisfactory results. Only two implants were lost in the “solely implant” group, whereas no implants failed in the “tooth-implant” group. No natural abutments were lost. In general, the biological and technical complications that did arise in both groups were minimal to moderate in severity and were easily managed. Survival and success rates of implants and ED-RDPs were comparable to those reported in the literature from university-based studies. Subjective masticatory function was rated high, and patients reported a very high OHRQoL. With regard to the aforementioned point, patients in the "solely implant" group exhibited a greater degree of satisfaction.

Thus, despite the demanding fabrication process, RDPs attached with electroplated double-crowns can represent a reliable and patient-specific solution.

## Data Availability

The data supporting the results of this study are not publicly available for reasons of privacy and are available from the corresponding author upon reasonable request.

## Abbreviations

ED-RDP: Electroplated double-crown removable dental prosthesis
OHIP: Oral Health Impact Profile
OHRQoL: Oral health-related quality of life
